# Blood pressure response to commonly administered antihypertensives for severe inpatient hypertension

**DOI:** 10.1371/journal.pone.0265497

**Published:** 2022-04-06

**Authors:** Lama Ghazi, Fan Li, Xinyuan Chen, Michael Simonov, Yu Yamamoto, Aditya Biswas, Jonathan Hanna, Tayyab Shah, Aldo J. Peixoto, F. Perry Wilson

**Affiliations:** 1 Department of Internal Medicine, Clinical and Translational Research Accelerator, Yale University, New Haven, CT, United States of America; 2 Department of Biostatistics, Yale School of Public Health, New Haven, CT, United States of America; 3 Department of Mathematics and Statistics, Mississippi State University, Mississippi State, MS, United States of America; 4 Department of Internal Medicine, Yale School of Medicine, Yale University, New Haven, CT, United States of America; 5 Department of Internal Medicine, Section of Nephrology, Yale School of Medicine, and the Hypertension Program, Yale New Haven Hospital Heart and Vascular Center, New Haven, CT, United States of America; Albert Einstein College of Medicine, UNITED STATES

## Abstract

**Background:**

Blood pressure (BP) elevations are commonly treated in hospitalized patients; however, treatment is not guideline directed. Our objective was to assess BP response to commonly prescribed antihypertensives after the development of severe inpatient hypertension (HTN).

**Methods:**

This is a cohort study of adults, excluding intensive care unit patients, within a single healthcare system admitted for reasons other than HTN who developed severe HTN (systolic BP>180 or diastolic BP >110 mmHg at least 1 hour after admission). We identified the most commonly administered antihypertensives given within 6 hours of severe HTN (given to >10% of treated patients). We studied the association of treatment with each antihypertensive vs. no treatment on BP change in the 6 hours following severe HTN development using mixed-effects model after adjusting for demographics and clinical characteristics.

**Results:**

Among 23,147 patients who developed severe HTN, 9,166 received antihypertensive treatment. The most common antihypertensives given were oral metoprolol (n = 1991), oral amlodipine (n = 1812), oral carvedilol (n = 1116), IV hydralazine (n = 1069) and oral hydralazine (n = 953). In the fully adjusted model, treatment with IV hydralazine led to 13 [-15.9, -10.1], 18 [-22.2, -14] and 11 [-14.1, -8.3] mmHg lower MAP, SBP, and DBP in the 6 hours following severe HTN development compared to no treatment. Treatment with oral hydralazine and oral carvedilol also resulted in significantly lower BPs in the 6 hours following severe HTN development (6 [-9.1, -2.1 and -7 [-9.1, -4.2] lower MAP, respectively) compared to no treatment. Receiving metoprolol and amlodipine did not result in a drop in BP compared to no treatment.

**Conclusion:**

Among commonly used antihypertensives, IV hydralazine resulted in the most significant drop in BP following severe HTN, while metoprolol and amlodipine did not lower BP. Further research to assess the effect of treatment on clinical outcomes and if needed which antihypertensives to administer are necessary.

## Introduction

Hypertension (HTN) is common in the inpatient setting with prevalence rate up to 72% [1]. Admission to the hospital due solely to HTN and its direct complications (hypertensive urgency and emergency) represents the minority of cases of inpatient blood pressure (BP) elevation (2/1000 adult emergency department visits, 0.75% of hospital admissions); far more common is HTN occurring after admission [2–4]. Additionally, treatment of HTN in the inpatient setting is not directed by guidelines. Physicians often treat asymptomatic inpatient HTN using intravenous (IV) antihypertensive agents although they might be unnecessary and could be harmful due to unpredictable BP reductions [5–7]. Additionally, oral antihypertensives have been shown to be equally associated with harm [4].

Severe HTN (systolic BP [SBP] / diastolic BP [DBP] >180/110 mmHg) may lead to acute injury to the heart, brain, kidney, and microvasculature and is associated with increased long-term cardiovascular disease complications [8–12]. Treatment is recommended for patients who are admitted for severe HTN with end organ damage (i.e. hypertensive emergency) to lower the BP and limit progressive injury; however there is paucity of data on treatment of those who develop severe HTN during hospitalization [4, 13].

We recently found that among patients hospitalized for reasons other than HTN at a large multi-hospital center, severe HTN was prevalent in 10% of patients and 40% received antihypertensive treatment, primarily oral antihypertensives [14]. Additionally, we report that excessive BP reduction (drop ≥30%) within 6 hours after severe HTN development was observed in treated and untreated patients. However, patients treated with IV antihypertensives compared to untreated or those treated with oral antihypertensives, had greater rates of acute severe BP reduction (i.e. within 6 hours of severe BP elevation). Given the clinical harms associated with severe and unpredictable BP reduction [8, 9, 13, 15–17], our goal was to characterize classes and types of antihypertensives that were commonly administered following severe HTN development. Additionally, we wanted to study the association of each of these antihypertensives with BP change using data from five teaching hospitals in Connecticut.

## Methods

### Study population

**Fig 1:**
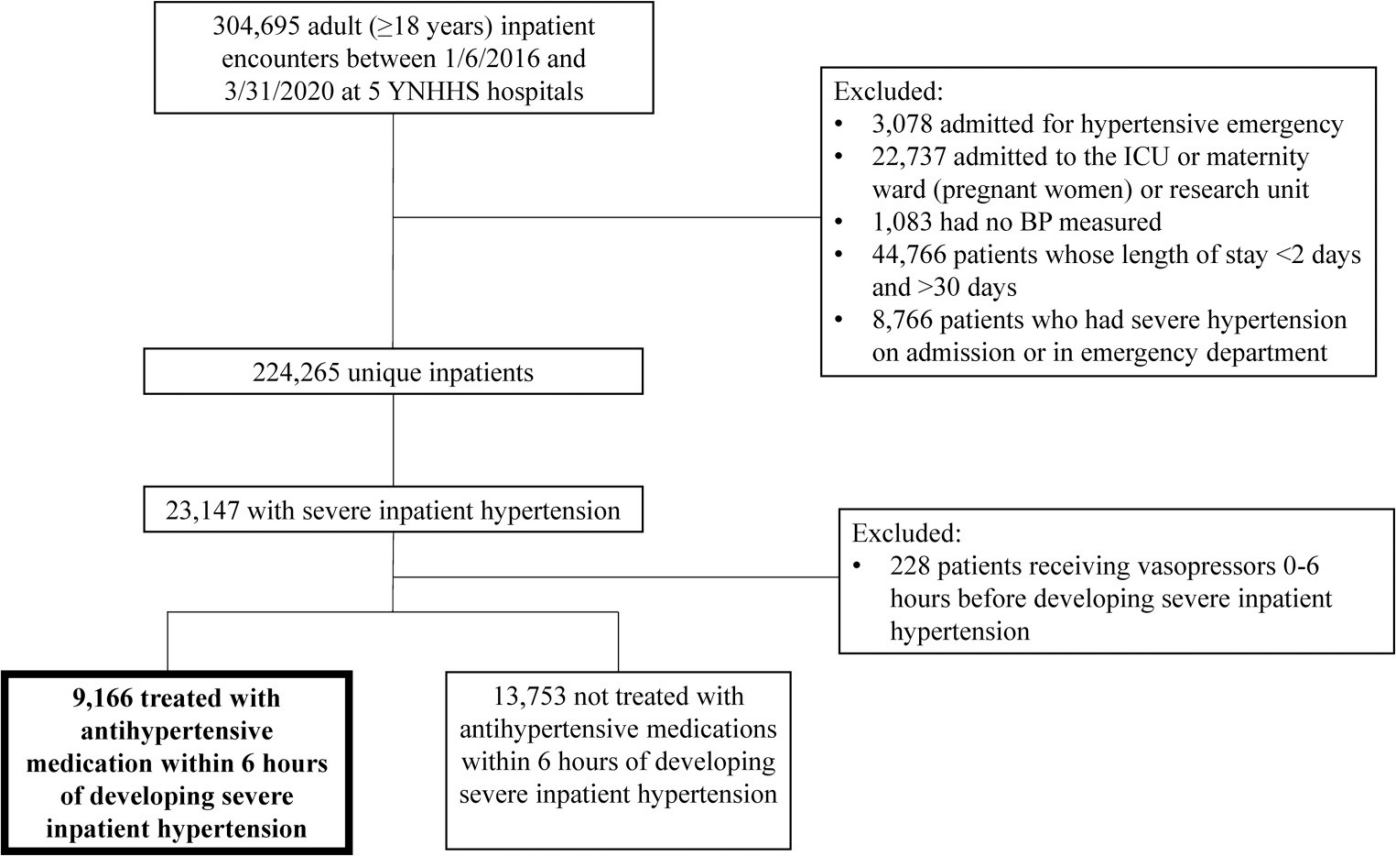
Study flow diagram. YNHHS: Yale New Haven Health System; ICU: intensive care unit; BP: blood pressure.

We included adult patients admitted to one of the five Yale New Haven Health System (YNHHS) hospitals between 1/6/2016 and 3/31/2020 (**Fig 1**). We excluded the following: 1) patients admitted for hypertensive emergency, urgency or crisis (**S1 File**); 2) pregnant women; 3) patients admitted to the intensive care unit; 4) patients admitted to the research unit; 4) patients who received vasopressors 0–6 hours before development of severe inpatient HTN (expanded on below). We did not collect data on patients who opted out of medical record research (less than 1% of all admissions). Among patients with multiple admissions with severe hypertension, we only included data from the first admission where severe hypertension occurred. This study was approved by the Yale Human Investigation Committee (HIC #2000028801) and need for consent was waived. Electronic health record data (EHR) was collected from the YNNHS data warehouse (EPIC, Verona WI).

### Defining severe inpatient HTN

We defined severe inpatient HTN as the first documentation of severe BP elevation (SBP>180 or DBP >110 mmHg) reported at least one hour after admission. We excluded all BPs captured in the emergency department. To avoid capturing falsely elevated BP measurements, we excluded patients whose BP dropped (SBP <180 and DBP<110 mmHg) within one hour of severe BP elevation and had no antihypertensive medications given during this time. If no repeat BP was measured, we considered the patient to have severe HTN.

### Identifying common antihypertensives

We captured antihypertensive medications, class and route given within 6 hours of severe HTN development. Once a patient received an antihypertensive, we considered them to be on that medication for the remainder of the 6 hour interval. An antihypertensive was considered commonly given if administered to ≥10% of treated patients.

### Covariates

We extracted demographics, vital signs, body mass index, comorbidities, medications and laboratory results from the EHR. Race and ethnicity were extracted from the self-reported demographic information and were included as both have been independently associated with BP.(8) Mean Arterial Pressure (MAP) was calculated using the following formula: 1/3 SBP + 2/3 DBP. We accounted for BP variability by including the coefficient of variation for MAP, SBP, and DBP (standard deviation of BP/mean BP of BPs obtained before severe HTN development). Comorbidities were assessed prior to admission and were defined per the Elixhauser comorbidity index using international classification of diseases (ICD)-10 codes and we calculated the total Elixhauser comorbidity score [18]. We captured information on the administration of narcotics, sedatives, benzodiazepines, non-steroidal anti-inflammatory (NSAID), corticosteroids, and crystalloid IV fluids administration as well as these agents may affect BP. We manually reviewed 100 charts to confirm BP measurements, medications and other covariates and found no inaccuracy compared to electronically-collected data.

### Outcome

Our primary outcome was BP (MAP, SBP, DBP) change over 6 hours following the development of severe HTN. A 6 hour interval reflects rapid changes in BP and that is of particular interest given the association of acute BP reductions with increased risk of death, damage to the vascular beds and cerebral hypoperfusion [8, 9, 13, 15–17].

### Statistical analysis

We described the characteristics of patients with severe HTN who were treated and untreated using χ^2^ test for proportions and Wilcoxon rank sum test for continuous variables. Frequency of each of the antihypertensive medications was obtained. Following that we identified the most common antihypertensives administered and presented descriptive characteristics for each group. Patients were not considered exposed to the antihypertensive until the time within the six hour window when the first dose was administered.

In order to quantify the association of antihypertensives on BP, we first aligned the patient-level data within each hour interval and fitted a linear mixed-effects model with a random intercept at the patient level and a random slope for time effect. This modeling strategy allows us to treat individuals as untreated until such time as they are initially treated, after which time they contribute data to the treated group, avoiding lead- and immortal-time bias [19–21]. With the aligned data, we obtain the association of treatment with each antihypertensive vs. no treatment on BP change by estimating the regression coefficients for each antihypertensive, with their 95% confidence intervals. We fit the following models: 1) unadjusted; 2) reduced adjusted model [additionally with covariates previously shown to be confounders and having a p-value ≤0.05] [8, 13, 22–26]. We adjusted for demographics, comorbidities associated with HTN, ward, baseline laboratory values, and relevant medications that influence BP administered 0–6 hours before onset of HTN; and 3) fully adjusted model (all covariates i.e. we further adjusted for additional comorbidities, BP variability, minimum and maximum BP recorded by patients before severe HTN development).

### Sensitivity analysis

We conducted several sensitivity analyses. First, we excluded patients who were admitted to the surgical ward, as treatment of these patients might defer from those in the medical ward. Second, in order to capture the association of de novo antihypertensive therapy in the hospital on BP response, we defined treatment as receiving a novel antihypertensive medication (a medication not prescribed for ≥90% of their hospital stay prior to the development of severe HTN). Third, we exclude patients that were cardiovascular admissions (**S1 File)** as antihypertensive medications are required in the patients [27–29]. Finally, irrespective of frequency of administration, we specifically assessed the BP response to labetalol and hydralazine given that both are guideline recommended to treat hypertensive emergency and are commonly administered in the United States [8, 13].

All analyses were performed using R (Vesion 4.00, Vienna Austria) [30]. This study utilized the Strengthening the Reporting of Observation Studies in Epidemiology (STROBE) guidelines.

## Results

### Cohort characteristics

There were 304,995 patients hospitalized between 1/2016 and 3/2020 in the YNHHS (**Fig 1**). We identified 224,265 eligible inpatients who were admitted for reasons other than HTN and of these 23,147 (10.3%) developed severe HTN. A total of 9,166 (39.6%) patients with severe HTN received antihypertensives within 6 hours of severe BP elevation (>180/110). Time from admission to first recorded severe BP elevation was 8 [1.3, 49.4] hours overall, 10 [2.2, 53.1] hours among those treated and 7 [1.0, 47.6] among those untreated (p<0.001). Treated patients compared to untreated were older, more likely to be Black, have greater number of comorbidities (Elixhauser score: 7 [4, 10] for treated vs. 6 [3, 9] for untreated; all p<0.001]. Treated and untreated patients had similar MAP at time of severe inpatient HTN diagnosis (121.7 vs. 122.3 mmHg) (**Table 1**).

**Table 1:** Baseline characteristics of patients treated and untreated within 6 hours of developing severe inpatient hypertension.

	Treated within 6 hours	Not treated within 6 hours	P-value
	N = 9,166 (40%)	N = 13,753 (60%)	
**Demographics**			
Age, years	73.0 (15.4)	70.4 (16.9)	<0.001
Male	4,068 (44.4)	6,119 (44.5)	0.879
Black	2,014 (22.0)	2,521 (18.3)	<0.001
Hispanic or Latino	909 (9.9)	1,413 (10.3)	0.292
*Service admitted to*			
Medical	7,738 (84.4)	11,135 (81.0)	<0.001
Surgical	1,428 (15.6)	2,618 (19.0)	
CVD admissions*	4,492 (49.0)	4,495 (32.7)	<0.001
**Comorbidities**			
Congestive Heart Failure	3,512 (38.3)	3,587 (26.1)	<0.001
Cardiac Arrhythmia	4,174 (45.5)	5,291 (38.5)	<0.001
Valvular Disease	2,283 (24.9)	2,649 (19.3)	<0.001
Pulmonary Circulation Disorder	1,307 (14.3)	1,498 (10.9)	<0.001
Peripheral Vascular Disease	2,735 (29.8)	3,242 (23.6)	<0.001
Hypertension	7,838 (85.5)	10,498 (76.3)	<0.001
Paralysis	455 (5.0)	643 (4.7)	0.332
Other Neurological Disorder	2,660 (29.0)	3,725 (27.1)	0.001
Chronic Pulmonary Disorders	3,578 (39.0)	4,922 (35.8)	<0.001
Diabetes	4,383 (47.8)	5,337 (38.8)	<0.001
Hypothyroidism	2,122 (23.2)	3,054 (22.2)	0.098
Renal Failure	3,591 (39.2)	3,921 (28.5)	<0.001
Liver Disease	1,285 (14.0)	2,066 (15.0)	0.037
Peptic Ulcer Disease (no bleeding)	586 (6.4)	671 (4.9)	<0.001
AIDS/HIV	123 (1.3)	221 (1.6)	0.119
Malignancy	1,792 (19.6)	2,766 (20.1)	0.305
Rheumatoid Arthritis/Collagen Disorders	763 (8.3)	1,116 (8.1)	0.588
Coagulopathy	1,247 (13.6)	1,849 (13.4)	0.743
Obesity	2,496 (27.2)	3,305 (24.0)	<0.001
Weight Loss	1,709 (18.6)	2,495 (18.1)	0.343
Fluid and Electrolyte Disorders	5,203 (56.8)	6,927 (50.4)	<0.001
Blood Loss Anemia	781 (8.5)	950 (6.9)	<0.001
Iron Deficiency Anemia	2,148 (23.4)	2,781 (20.2)	<0.001
Alcohol Use Disorder	1,1194 (13.0)	1,820 (13.2)	0.664
Drug Abuse	1,256 (13.7)	1,953 (14.2)	0.296
Psychosis	529 (5.8)	876 (6.4)	0.069
Depression	3,210 (35.0)	4,542 (33.0)	0.002
*Elixhauser Score*	7 [4, 10]	6 [3, 9]	<0.001
**Admission Characteristics, Median [IQR]**			
MAP	105.0 [92.7, 116.3]	106.0 [93.3, 117.7]	<0.001
SBP (mmHg)	154.0 [135.0, 172.0]	153.0 [134.0, 173.0]	0.176
DBP (mmHg)	80.0 [69.0, 91.0]	81.0 [70.0, 92.0]	<0.001
Heart Rate (bpm)	81.0 [70.0, 95.0]	83.0 [71.0, 98.0]	<0.001
BMI (kg/m^2^)	27.5 [23.3, 32.6]	27.3 [23.2, 32.4]	0.030
**Coefficient of variation (CV) of BP and MAP before onset of severe inpatient hypertension (SD/mean)**			
MAP CV	0.10 [0.07, 0.13]	0.10 [0.07, 0.13]	0.485
SBP CV	0.11 [0.07, 0.14]	0.10 [0.07, 0.14]	0.090
DBP CV	0.12 [0.09, 0.16]	0.12 [0.09, 0.16]	0.225
**BP at time of incident severe inpatient hypertension**			
MAP	121.7 [115.0, 129.0]	122.3 [115.3, 129.0]	0.038
SBP	186.0 [182.0, 192.0]	184.0 [181.0, 189.0]	<0.001
DBP	88.0 [79.0, 101.0]	90.0 [80.0, 111.0]	<0.001
**Admission Laboratory Values Median [IQR]**			
Serum Sodium (meq/L)	139.0 [136.0, 141.0]	139.0 [136.0, 141.0]	0.311
Serum Potassium (meq/L)	4.2 [3.8, 4.6]	4.2 [3.8, 4.5]	<0.001
Serum Chloride (meq/L)	102.0 [98.0, 105.0]	102.0 [98.0, 105.0]	0.248
Serum Bicarbonate (meq/L)	25.0 [22.0, 27.0]	24.5 [22.0, 27.0]	0.534
BUN (mg/dL)	22.0 [15.0, 35.0]	20.0 [14.0, 30.0]	<0.001
Serum Creatinine (mg/dL)	1.1 [0.8, 1.8]	1.0 [0.8, 1.5]	<0.001
eGFR (ml/min/1.73m2)	56.7 [33.0, 83.9]	67.9 [42.2, 96.7]	<0.001
White Blood Cell Count (x1000/uL)	8.8 [6.6, 11.7]	9.1 [6.8, 12.3]	<0.001
Platelet Count (x1000/uL)	223.0 [175.0, 282.0]	225.0 [174.0, 285.0]	0.213
Hemoglobin, g/dl	11.8 [10.2, 13.3]	12.1 [10.6, 13.6]	<0.001
Hematocrit, %	36.5 [32.0, 40.8]	37.4 [33.0, 41.4]	<0.001
**Received any of the following medications 0–6 hours before time of incident severe inpatient hypertension**			
Steroids	348 (3.8)	627 (4.6)	0.006
NSAID	153 (1.7)	338 (2.5)	<0.001
Crystalloid	1,272 (13.9)	2,112 (15.4)	0.002
Narcotic	1,303 (14.2)	2,252 (16.4)	<0.001
Sedatives	482 (5.3)	1,069 (7.8)	<0.001

Values are presented as count (percent) or median (IQR). BMI: body mass index, BP: blood pressure, MAP: mean arterial pressure; SBP: systolic BP; DBP: diastolic BP; bpm: beats per minute, BUN: Blood Urea Nitrogen. eGFR: Estimated glomerular filtration rate

Steroids: prednisone, methylprednisolone, ketorolac, hydrocortisone, dexamethasone, budesonide

NSAIDS: ketorolac, piroxicam, oxaprozin, diclofenac, indomethacin, naproxen, ibuprofen, celecoxib

Crystalloids: ringers drip, ringers bolus, saline drip, saline bolus

Narcotics: oxymorphone, tramadol, remifentanil, oxycodone, morphine, methadone, meperidine, hydromorphone, hydrocodone, fentanyl, buprenorphine

Sedatives: midazolam, lorazepam, diazepam, clonazepam, alprazolam

* CVD (cardiovascular) admissions: cerebrovascular accident, myocardial infarction, heart failure or abdominal aortic aneurysm (based on the following ICD-10 codes: I60, I61, I63, I64, G45, I62, I66, I67, I68, G46, I-21, I22, I25.2, I71).

### Common antihypertensives

Of those who received treatment within 6 hours of severe inpatient HTN development, the median [interquartile range] number of antihypertensive medications given was 1 [1, 1.75]. Beta-blockers were the most commonly administered antihypertensive class (**S1 Table in S1 File**). *Metoprolol (oral)*,*amlodipine (oral)*, *carvedilol (oral)*, *and hydralazine (IV and oral)* were the most common antihypertensives given within 6 hours of developing severe inpatient HTN and our analyses will focus on these medications (**S2 Table in S1 File**). Characteristics of patients receiving each of the common antihypertensives can be found in **S3** and **S4 Tables in S1 File**. Patients treated with oral metoprolol were on average 76 years of age, 44% were male, commonly had history of HTN (87%) and cardiac arrythmias (55%) and diabetes (45%), median admission BP was 148/78, and median BP at time of severe HTN was 185/88. Those treated with oral amlodipine were on average 72 years of age, commonly had a history of HTN (83%) and diabetes, (41%), median admission BP was 152/79 and median BP at time of severe HTN was 185/88. As for patients given oral carvedilol, mean age was 72, 64% were cardiovascular disease admissions, commonly had a history of HTN (83%) and diabetes (41%), median eGFR on admission was 42 [20, 67] ml//min/1.73 m^2^, median BP on admission was 148/75 and median BP at time of severe HTN development was 185/85. Those treated with IV hydralazine were on average 71 years, had a history of HTN (80%) and diabetes (42%), median BP on admission was 160/83 and median BP at time of severe HTN was 188/90. Finally, for patients treated with IV hydralazine, average age was 72, commonly had a history of HTN (91%) and diabetes (57%), and median eGFR on admission was 35 [30, 39] ml//min/1.73 m^2^, median BP on admission was 156/78 and median BP at time of severe HTN development was 186/86. Overall, all patients regardless of antihypertensive class they received had a history of HTN and diabetes and similar BPs at time of severe inpatient HTN development. However, patients treated with IV hydralazine had the highest admission BPs and those treated with carvedilol were more likely to be admitted for cardiovascular reasons. Moreover, kidney function, as measured by estimated Glomerular Filtration rate(32, 33), was lowest among those treated with oral hydralazine and oral carvedilol.

### Quantifying the dynamic effect of common antihypertensives on BP

**Fig 2:**
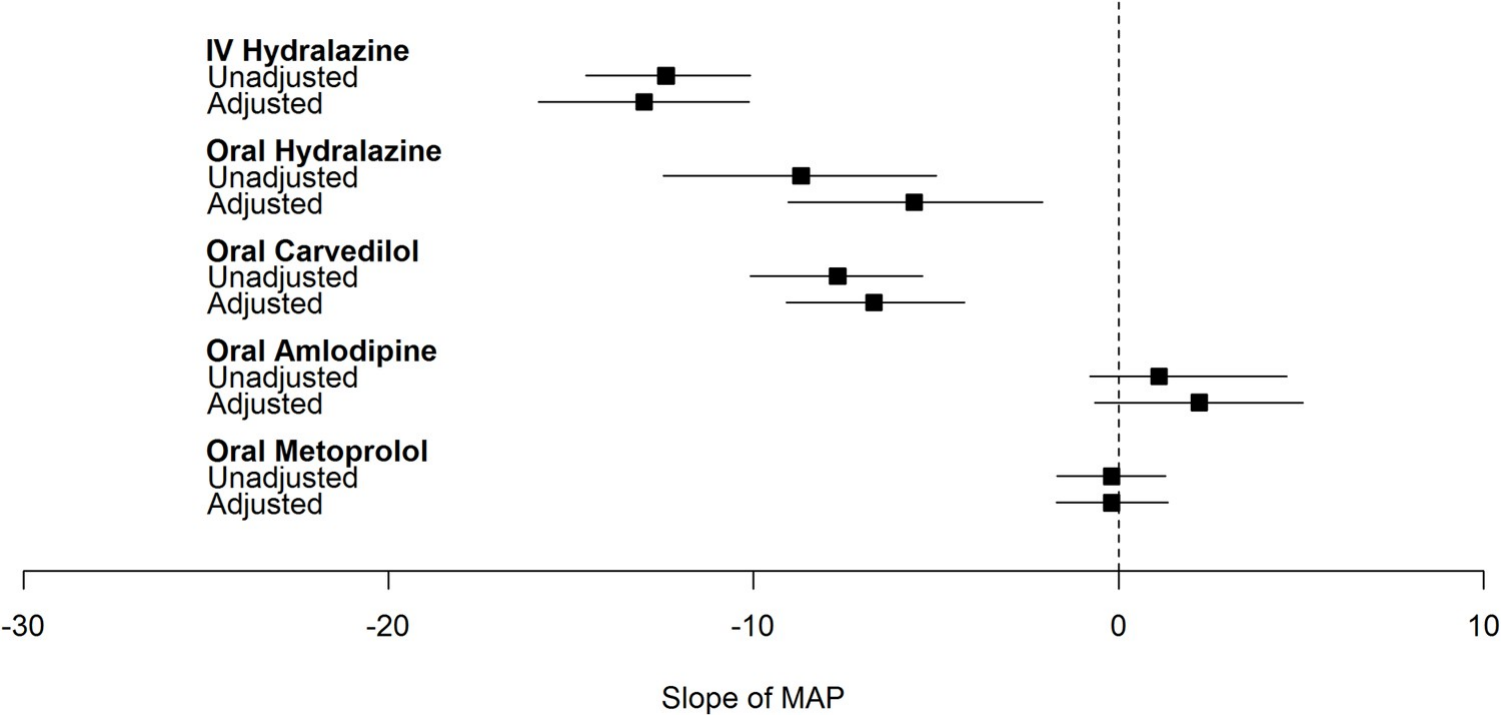
Plot of effect estimate (β [95%CI]) of common antihypertensives on slope of mean arterial pressure within 6 hours of severe inpatient hypertension development. Adjusted for age, sex, race, ethnicity, ward, comorbidities (congestive heart failure, cardiac arrythmia, valvular disease, pulmonary circulation disorder, peripheral vascular disease, hypertension, paralysis, other neurological disorders, chronic pulmonary disease, diabetes, hypothyroidism, renal failure, liver disease, peptic ulcer disease excluding bleeding, AIDS/HIV, lymphoma, cancer, rheumatoid arthritis/collagen disorder, coagulopathy, obesity, weight loss, fluid and electrolyte disorders, blood loss anemia, deficiency anemia, alcohol abuse, drug abuse, psychosis, depression), baseline laboratory values (sodium, potassium, chloride, bicarbonate, BUN, eGFR, WBCC, platelet count, hemoglobin, hematocrit), NSAID use 0–6 hours before time of severe inpatient HTN, crystalloid use 0–6 hours before time of severe inpatient HTN, steroid use 0–6 hours before time of severe inpatient HTN, narcotic use 0–6 hours before time of severe inpatient HTN, sedative use 0–6 hours before time of severe inpatient HTN, maximum MAP before time of severe inpatient HTN development, minimum MAP before time of severe inpatient HTN development, coefficient of variation of MAP before time of severe inpatient HTN development. Hospital.

**Fig 3:**
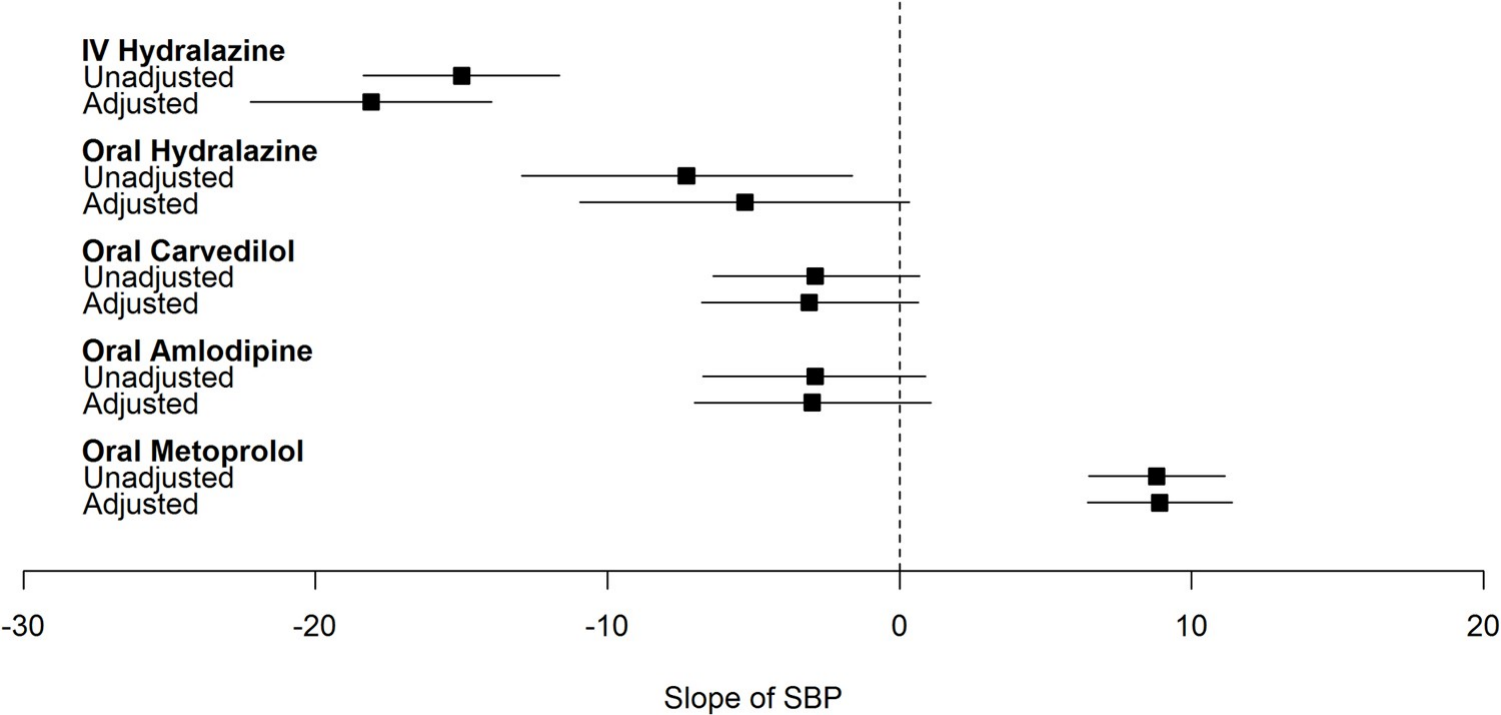
Plot of effect estimate (β [95%CI]) of common antihypertensives on slope of systolic blood pressure within 6 hours of severe inpatient hypertension development. Adjusted for age, sex, race, ethnicity, ward, comorbidities (congestive heart failure, cardiac arrythmia, valvular disease, pulmonary circulation disorder, peripheral vascular disease, hypertension, paralysis, other neurological disorders, chronic pulmonary disease, diabetes, hypothyroidism, renal failure, liver disease, peptic ulcer disease excluding bleeding, AIDS/HIV, lymphoma, cancer, rheumatoid arthritis/collagen disorder, coagulopathy, obesity, weight loss, fluid and electrolyte disorders, blood loss anemia, deficiency anemia, alcohol abuse, drug abuse, psychosis, depression), baseline laboratory values (sodium, potassium, chloride, bicarbonate, BUN, eGFR, WBCC, platelet count, hemoglobin, hematocrit), NSAID use 0–6 hours before time of severe inpatient HTN, crystalloid use 0–6 hours before time of severe inpatient HTN, steroid use 0–6 hours before time of severe inpatient HTN, narcotic use 0–6 hours before time of severe inpatient HTN, sedative use 0–6 hours before time of severe inpatient HTN, maximum MAP before time of severe inpatient HTN development, minimum MAP before time of severe inpatient HTN development, coefficient of variation of MAP before time of severe inpatient HTN development. hospital.

**Fig 4:**
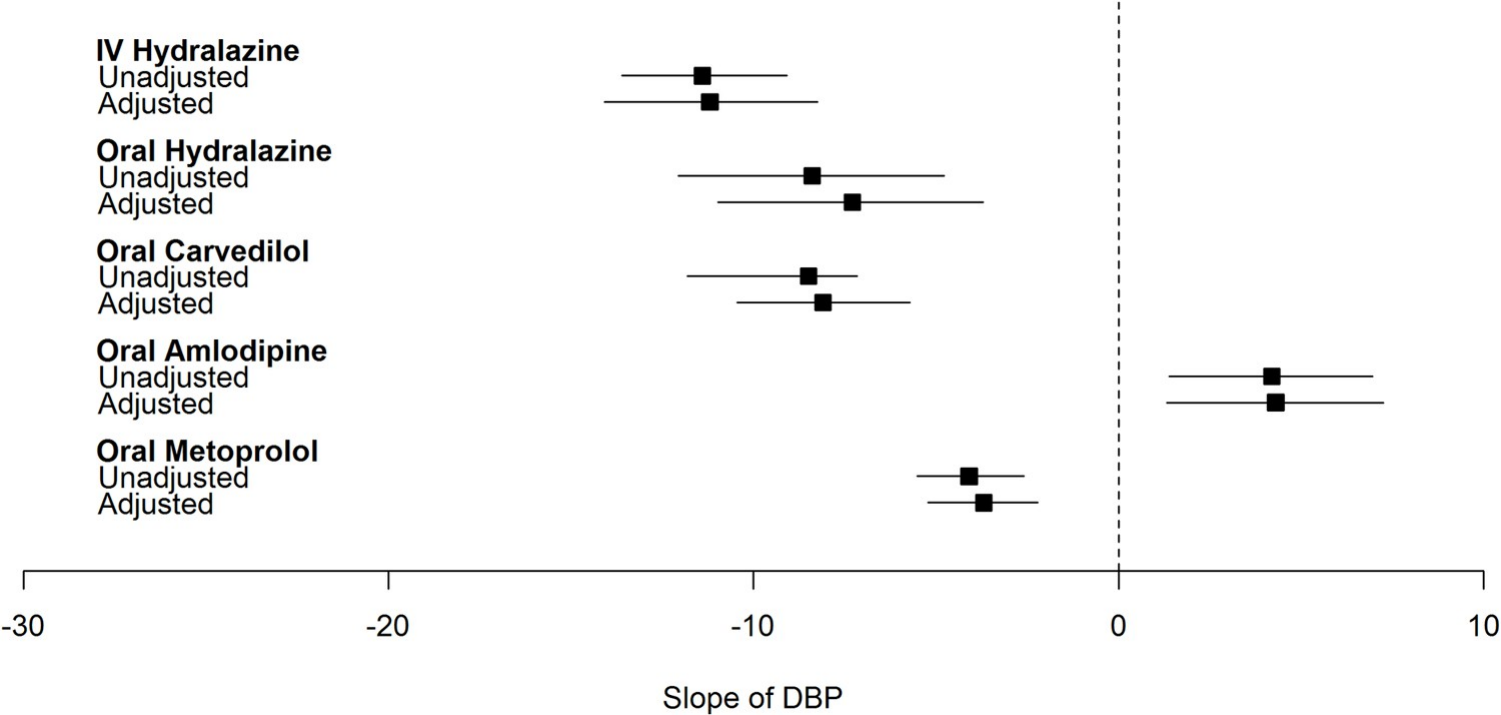
Plot of effect estimate (β [95%CI]) of common antihypertensives on slope of diastolic blood pressure within 6 hours of severe inpatient hypertension development. Adjusted for age, sex, race, ethnicity, ward, comorbidities (congestive heart failure, cardiac arrythmia, valvular disease, pulmonary circulation disorder, peripheral vascular disease, hypertension, paralysis, other neurological disorders, chronic pulmonary disease, diabetes, hypothyroidism, renal failure, liver disease, peptic ulcer disease excluding bleeding, AIDS/HIV, lymphoma, cancer, rheumatoid arthritis/collagen disorder, coagulopathy, obesity, weight loss, fluid and electrolyte disorders, blood loss anemia, deficiency anemia, alcohol abuse, drug abuse, psychosis, depression), baseline laboratory values (sodium, potassium, chloride, bicarbonate, BUN, eGFR, WBCC, platelet count, hemoglobin, hematocrit), NSAID use 0–6 hours before time of severe inpatient HTN, crystalloid use 0–6 hours before time of severe inpatient HTN, steroid use 0–6 hours before time of severe inpatient HTN, narcotic use 0–6 hours before time of severe inpatient HTN, sedative use 0–6 hours before time of severe inpatient HTN, maximum MAP before time of severe inpatient HTN development, minimum MAP before time of severe inpatient HTN development, coefficient of variation of MAP before time of severe inpatient HTN development. hospital.

Overall, in the fully adjusted models, treatment with IV and oral hydralazine, and oral carvedilol resulted in significantly lower MAP in the 6 hours following severe inpatient HTN development (**Figs 2–4**). The greatest magnitude in BP change was observed with IV hydralazine. On the other hand, oral metoprolol was associated with significant decrease in DBP only and amlodipine resulted in either no change or an increase in BP following administration.

Specifically, in the fully adjusted model treatment with IV hydralazine led to 13 [-15.9, -10.1], 18 [-22.2, -14] and 11 [-14.1, -8.3] mmHg lower MAP, SBP, and DBP in the 6 hours following severe inpatient HTN development (**Table 2**). Similarly, treatment with oral hydralazine led to lower BPs but with a smaller magnitude of change when compared with IV hydralazine. For example, oral hydralazine was associated with a 5.6 mmHg lower MAP compared to a 13 mmHg drop in MAP observed with IV hydralazine. Oral carvedilol decreased MAP, SBP and DBP by 7, 3 and 8 mmHg respectively in the fully adjusted model. Of note, MAP response following metoprolol administration was minimal (-0.2 mmHg).

**Table 2:** Association of the most common antihypertensives given for treatment of severe inpatient hypertension with blood pressure.

β (95%CI)	Model 1	Model 2	Model 3
**Treated with oral metoprolol vs. untreated**			
Slope of MAP	-0.2 [-1.69, 1.28]	0.3 [-1.24, 1.76]	-0.2 [-1.71, 1.35]
Slope of SBP	8.8 [6.47, 11.14]	8.7 [6.35, 11.09]	8.9 [6.44, 11.39]
Slope of DBP	-4.1 [-5.52, -2.59]	-3.1 [-4.55, -1.63]	-3.7 [-5.22, -2.22]
**Treated with oral amlodipine vs. untreated**			
Slope of MAP	1.1 [-0.79, 4.60]	2.0 [-0.76, 4.79]	2.2 [-0.65, 5.05]
Slope of SBP	-2.9 [-6.74, 0.88]	-3.5 [-7.41, 0.47]	-3.0 [-7.02, 1.08]
Slope of DBP	4.2 [1.38, 6.96]	4.6 [1.75, 7.39]	4.3 [1.31, 7.26]
**Treated with oral carvedilol vs. untreated**			
Slope of MAP	-7.7 [-10.09, -5.37]	-7.2 [-9.65, -4.83]	-6.7 [-9.11, -4.22]
Slope of SBP	-2.9 [-6.39, 0.69]	-3.9 [-7.52, -2.12]	-3.1 [-6.77, 0.65]
Slope of DBP	-8.5 [-11.82, -7.17]	-8.4 [-10.72, -6.08]	-8.1 [-10.46, -5.72]
**Treated with IV hydralazine vs. untreated**			
Slope of MAP	-12.4 [-14.69, -10.09]	-12.1 [-14.48, -9.79]	-13.0 [-15.89, -10.12]
Slope of SBP	-15.0 [-18.37, -11.64]	-15.4 [-18.81, -11.90]	-18.1 [-22.23, -13.97]
Slope of DBP	-11.4 [-13.61, -9.08]	-11.2 [-13.50, -8.89]	-11.2 [-14.09, -8.25]
**Treated with oral hydralazine vs. untreated**			
Slope of MAP	-8.7 [-12.48, -4.99]	-8.1 [-11.85, -4.33]	-5.6 [-9.05, -2.08]
Slope of SBP	-7.3 [-12.95, -1.63]	-7.7 [-13.43, -1.93]	-5.3 [-10.94, 0.34]
Slope of DBP	-8.4 [-12.06, -4.78]	-7.6 [-11.26, -3.99]	-7.3 [-10.97, -3.71]

BP: blood pressure; IV: intravenous; MAP: mean arterial pressure; SBP: systolic blood pressure; DBP: diastolic blood pressure

Within 6 hours of developing severe inpatient HTN, number of patients that were treated with only: oral metoprolol: 998; oral amlodipine: 659; oral carvedilol:473; IV hydralazine: 632; oral hydralazine: 390 vs. untreated (13,753)

**Model 1**: unadjusted

**Model 2**: age, sex, race, ethnicity, ward, comorbidities (congestive heart failure, cardiac arrythmia, peripheral vascular disease, hypertension, diabetes, hypothyroidism, renal failure, AIDS/HIV, cancer, alcohol abuse, drug abuse, psychosis, depression), baseline laboratory values (sodium, potassium, chloride, bicarbonate, BUN, eGFR, WBCC, platelet count, hemoglobin, hematocrit), NSAID use 0–6 hours before time of severe inpatient HTN, crystalloid use 0–6 hours before time of severe inpatient HTN, steroid use 0–6 hours before time of severe inpatient HTN, narcotic use 0–6 hours before time of severe inpatient HTN, sedative use 0–6 hours before time of severe inpatient HTN, hospital

**Model 3**: age, sex, race, ethnicity, ward, comorbidities (congestive heart failure, cardiac arrythmia, valvular disease, pulmonary circulation disorder, peripheral vascular disease, hypertension, paralysis, other neurological disorders, chronic pulmonary disease, diabetes, hypothyroidism, renal failure, liver disease, peptic ulcer disease excluding bleeding, AIDS/HIV, lymphoma, cancer, rheumatoid arthritis/collagen disorder, coagulopathy, obesity, weight loss, fluid and electrolyte disorders, blood loss anemia, deficiency anemia, alcohol abuse, drug abuse, psychosis, depression), baseline laboratory values (sodium, potassium, chloride, bicarbonate, BUN, eGFR, WBCC, platelet count, hemoglobin, hematocrit), NSAID use 0–6 hours before time of severe inpatient HTN, crystalloid use 0–6 hours before time of severe inpatient HTN, steroid use 0–6 hours before time of severe inpatient HTN, narcotic use 0–6 hours before time of severe inpatient HTN, sedative use 0–6 hours before time of severe inpatient HTN, maximum MAP before time of severe inpatient HTN development, minimum MAP before time of severe inpatient HTN development, coefficient of variation of MAP before time of severe inpatient HTN development.hospital.

We observed similar trends for IV and oral hydralazine in our *sensitivity analysis* (**S5-S7 Tables in S1 File**) when we considered only medical ward patients, or only de novo antihypertensives or among non cardiovascular disease admissions. We found consistently, that IV and oral hydralazine were associated with significant decrease in MAP, SBP and DBP. As prespecified, we additionally assessed BP response to the non-commonly administered antihypertensive labetalol. IV and oral labetalol were administered among 337 and 129 patients within 6 hours of developing severe HTN. In the fully adjusted model treatment with IV labetalol led to a 8, 16, and 6 mmHg drop in MAP, SBP, and DBP, respectively, compared to untreated patients (**S8 Table in S1 File**). Oral labetalol was not significantly associated with a drop in BP.

## Discussion

In this cohort study of patients hospitalized for reasons other than HTN, 9,166 (10% of hospitalized patients) developed severe HTN and 40% received antihypertensive treatment within 6 hours. The most common antihypertensives prescribed included oral metoprolol, oral amlodipine, oral carvedilol, IV and oral hydralazine. Patients with severe HTN who were treated with hydralazine, irrespective of treatment route, had a lower MAP, SBP and DBP compared to untreated inpatients. This was consistent across all sensitivity analyses and after adjustment for demographic and clinical characteristics. Overall, treatment with amlodipine and metoprolol did not lead to a lower MAP within 6 hours of severe HTN development.

Other studies have looked at antihypertensive treatment following elevated BP during hospitalization. The most common antihypertensives studied to manage severe BP elevation during hospitalization included as needed IV hydralazine and IV labetalol [5–7, 31]. These studies demonstrated that IV antihypertensives were generally administered to patients who do not meet the criteria of acute BP elevation (BP<160/110) and most patients were not continued on their home antihypertensives. Additionally, a recent study of 250 medical patients hospitalized at the University of Colorado between 2014 and 2016 assessed the use of “as needed” hydralazine and labetalol for treatment of asymptomatic HTN. They observed that 36% of patients were given antihypertensives for non-acute BP elevations (defined as BP <160/110) and that oral hydralazine was the most commonly administered antihypertensive [32]. In our study we found that hydralazine was also commonly prescribed following severe BP elevation (SBP>180 or DBP>110 mmHg). IV labetalol though not commonly administered in our cohort, also led to a significant drop in BP when compared to no treatment. The choice of medications reflects both real life preference and medications available in our institution’s formulary. Understanding which antihypertensives could lead to severe BP reduction is important, as future treatment guidelines will rely on effect of treatment on BP trajectory and clinical outcomes. Rapid reduction following severe asymptomatic BP elevation is associated with risk of adverse events if BP is lowered below the ability to autoregulate and maintain tissue perfusion [33–35]. Additionally, rapid BP reductions have no proven benefit [8, 9, 34, 36–38]. Therefore, in the future, choice of medications should rely on clinical evidence rather than preference or availability.

To our knowledge, there have been no previous studies assessing real-life antihypertensive preferences to treat severe HTN in hospitalized patients. However, some trials have assessed the effect of different antihypertensives in treating hypertensive emergency (admission to the hospital for severe HTN with end organ damage) and hypertensive urgency (admission to the hospital for severe HTN). Peixoto summarized the latest evidence in a recent review [13]. The recommendation was to start IV antihypertensives and pace BP lowering in the setting of hypertensive emergency and to use oral guideline-concordant long acting antihypertensives for hypertensive urgency. Clonidine and labetalol were recommended for hypertensive urgency as they may result in less abrupt BP changes [39–41]. Hydralazine use was discouraged as it has unpredictable pharmacokinetics and can lead to excessive BP lowering and increased risk of reflex tachycardia [5, 8, 9, 42, 43]. Despite all the evidence recommending avoiding using hydralazine both IV or oral to treat severe HTN development, it still remains one of the most common antihypertensives used across different health systems and studies [5–7, 31].

In our study of patients admitted for reasons other than HTN, we observed an overall fall of 8.5 to 13 mmHg and 5.6 to 9.9 mmHg in MAP following IV hydralazine and oral hydralazine administration, respectively, even after excluding patients admitted to the surgical ward, excluding cardiovascular admissions and only assessing the effect of a new medication order. Additionally, IV labetalol resulted in a significant decrease in BP compared to no treatment. On the other hand, we found that metoprolol and amlodipine did not result in lower MAP compared to no treated. When including only new orders of carvedilol, i.e. non-standing orders, we found that carvedilol does not result in MAP reduction. Additionally, the difference in beta-blockers (carvedilol and metoprolol) effect on SBP could be explained by the different mechanism in which they act. Metoprolol is associated with an increase in SBP, this might be due to the unopposed alpha-adrenergic activity leading to increase in sympathetic activation and stroke volume [44]. Carvedilol, however, results in lower SBP and this might be due to its alpha blocking [45]. This suggests that if treatment is needed following severe BP elevation among patients admitted for reasons other than HTN, effect of each antihypertensive class on BP following severe HTN development should be taken into consideration.

Other health systems have reported on quality initiative programs that were instituted to reduce the reliance on IV antihypertensives. In a single center cohort study of 2,306 hospitalized adults, including intensive care unit, episodes of severe HTN (BP >160/90) occurred an average of 9 times during hospitalization and 11% of patients received IV hydralazine, IV labetalol or both. This San Francisco center then conducted a quality improvement initiative consisting of an educational campaign and changing the EHR BP notification from 160/90 mmHg to 190/90 mmHg. Following this initiative, patients were 40% less likely to receive IV medications, however no change in patients’ median BP or outcomes (ICU transfers, cardiopulmonary arrests or rapid response calls) was observed [46]. A similar quality improvement study was conducted in New York to decrease the use of IV antihypertensives [47]. These studies suggest that evaluation of the underlying cause of elevated BP is necessary before beginning treatment and that oral antihypertensives are preferred to IV antihypertensives [48]. We concur with these studies that evaluation of the underlying cause of severe HTN is necessary, and treatment might be recommended in certain situations, though these are not clearly defined. However, in the absence of evidence to support acute treatment in patients with no acute target organ damage, our results indicate that hydralazine (IV or oral) may be used if BP reduction is desired. Metoprolol and amlodipine, on the other hand are not suitable for rapid reductions. Additional research is needed to explore which antihypertensives (class and dose) might be better suited to treat severe BP elevations.

Our study is subject to several limitations. First, we do not have data on home BP medications and class of home antihypertensive might affect clinician’s decision making. However, we were able to identify which antihypertensives were standing orders and which were administered for the first time following severe HTN development. Second, we relied on one BP measurement to define severe BP elevation. This might introduce bias as we may overestimate the prevalence of elevated BP. However, this misclassification will bias our results towards the null and we would have reported a more conservative estimate of the association of treatment with BP response. Third, confounders such as type of ward, reason for admission, and pain medications or anxiolytics might affect the response of antihypertensive medications on BP. We accounted for these confounders in our sensitivity analyses but we are still subject to residual and unknown confounders. Fourth, we did not assess BP dose response to each antihypertensive as this is beyond the scope of the analysis. Finally, given the complexity of treatment decisions and the lack of guidelines, we were unable to account for the decision making behind treating certain patients with specific antihypertensives and therefore we are unable to account for this selection bias. Our study has several strengths. We have a well characterized cohort and manually validated a subset of our data (96% sensitivity for all covariates). Additionally, we show that there is substantial practice variation in the treatment of severe HTN and this study is the first step to help establish standardized care for this patient population. Inconsistent care practices can lead to worse patient outcomes [49, 50] and guidelines are needed to standardize care to the benefit of all patients.

## Conclusions

We found that in a cohort of hospitalized patients admitted for reasons other than HTN, 10% had severe BP elevation of which 40% were treated with antihypertensives. Metoprolol, amlodipine, carvedilol and hydralazine were the most commonly administered antihypertensives within 6 hours of developing severe HTN. Hydralazine, IV and oral, compared to untreated inpatients resulted in a significant drop in MAP. This suggests that certain antihypertensives might be better suited to decrease BP following severe HTN development if indicated. Future studies should phenotype hospitalized patients with severe HTN and study the effect of treatment overall and by antihypertensive class on clinical outcomes (acute kidney injury, cardiovascular events, death). Subsequent trials may then compare the most effective antihypertensives to use if deemed necessary. This will be essential to help establish guidelines to treat severe HTN in hospitalized patients.

## Supporting information

S1 File (DOCX)

S1 Graphical abstract (TIF)

## Abbreviations

BP: blood pressure
DBP: diastolic blood pressure
EHR: electronic health record
HTN: hypertension
ICD: international classification of disease
IV: intravenous
MAP: mean arterial pressure
NSAID: non-steroidal anti-inflammatory
SBP: systolic blood pressure
YNHHS: Yale New Haven Health System
