# Supplementary material for: Blood pressure response to commonly administered antihypertensives for severe inpatient hypertension
Source: PLoS One. 2022 Apr 6;17(4):e0265497. doi: 10.1371/journal.pone.0265497 (PMC8985959; doi:10.1371/journal.pone.0265497)
Supplement: S1 File — (DOCX) [file pone.0265497.s001.docx]

**Supporting Information**

**Blood pressure response to commonly administered antihypertensives for severe inpatient hypertension**Lama Ghazi MD, PhD*^1^, Fan Li PhD^2^, Xinyuan Chen PhD^3^, Michael Simonov MD^1^, Yu Yamamoto MS^1^, Aditya Biswas MS^1^, Jonathan Hanna MD^4^, Tayyab Shah MD^4^, Aldo J. Peixoto, MD^¶5^  , F. Perry Wilson MD, MSCE^¶1^

^1^Department of Internal Medicine, Clinical and Translational Research Accelerator, Yale University, New Haven, CT

^2^Department of Biostatistics, Yale School of Public Health, New Haven, CT

^3^Department of Mathematics and Statistics, Mississippi State University, Mississippi State, MS

^4^Department of Internal Medicine, Yale School of Medicine, Yale University, New Haven, CT

^5^Department of Internal Medicine, Section of Nephrology, Yale School of Medicine, and the Hypertension Program, Yale New Haven Hospital Heart and Vascular Center, New Haven, CT

Additional Information:

**Relevant International Classification of Diseases (ICD-10) codes:**Hypertensive emergency, urgency or crisis: I16.0, I16.1, I16.9
Cardiovascular admissions i.e. have cerebrovascular accidents, myocardial infarction, heart failure, abdominal aortic aneurysm: I60, I61, I63, I64, G45, I62, I66, I67, I68, G46 , I-21, I22, I25.2, I71

Table S1. Common antihypertensive drug classes administered within 6 hours of developing severe inpatient hypertension

|  | Within 6 hours of developing severe inpatient hypertension, n (%) (#class/ #total incident severe hypertension*100%) |
| --- | --- |
| Angiotensin Converting Enzyme Inhibitors/ Angiotensin Receptor Blockers^1^ | 1,706 (7.4) |
| Calcium Channel Blockers^2^ | 2,405 (10.4) |
| Alpha antagonists^3^ | 123 (0.5) |
| Beta blockers^4^ | 4,575 (20.0) |
| Diuretics^5^ | 1,637 (7.1) |
| Renin Inhibitors^6^ | 0 (0) |
| Vasodilators^7^ | 2,110 (9.2) |
| None | 13,753 (60.0) |

# Total incident severe hypertension: 22,919. Patients might be on more than one antihypertensive class, therefore percentages do not add to a 100%

1. ACEi/ARB: benazepril, quinapril, ramipril, lisinopril, enaliprat, captopril, candesartan, telmisartan, olmesartan, valsartan, losartan, irbesartan
2. Calcium channel blockers: verapamil, nimodipine, nifedipine, nicardipine, diltiazem, amlodipine
3. Alpha antagonist: doxazosin, prazosin
4. Beta blockers: propranolol, nadolol, metoprolol, labetalol, esmolol, carvedilol, atenolol
5. Diuretics: spironolactone, hydrochlorothiazide, furosemide, eplerenone, chlorthalidone
6. Renin Inhibitors: aliskiren
7. Vasodilator: hydralazine, nitroglycerin, nitroprusside

Table S2. Antihypertensives given within 6 hours of developing severe inpatient hypertension

| **Antihypertensive medications given to inpatients within 6 hours of developing severe hypertension** | **Number** |
| --- | --- |
| Amiloride (oral) | 4 |
| **Amlodipine (oral)** | **1,812** |
| Atenolol (oral) | 168 |
| Captopril (oral) | 23 |
| **Carvedilol (oral)** | **1,116** |
| Chlorthalidone (oral) | 14 |
| Clonidine (oral) | 208 |
| Diltiazem | |
| Diltiazem (IV) | 84 |
| Diltiazem (oral) | 101 |
| Doxazosin (oral) | 101 |
| Enalapril (oral) | 2 |
| Enalaprilat (IV) | 35 |
| Eplerenone (other) | 1 |
| Esmolol (IV) | 8 |
| Furosemide | |
| Furosemide (IV) | 914 |
| Furosemide (oral) | 444 |
| Hydrochlorothiazide (oral) | 221 |
| **Hydralazine** | |
| **Hydralazine (IV)** | **1,069** |
| **Hydralazine (oral)** | **953** |
| Labetalol | |
| Labetalol (IV) | 611 |
| Labetalol (oral) | 324 |
| Lisinopril (oral) | 884 |
| Losartan (oral) | 740 |
| **Metoprolol** | |
| Metoprolol (IV) | 438 |
| **Metoprolol (oral)** | **1,991** |
| Minoxidil (oral) | 9 |
| Nadolol (oral) | 23 |
| Nicardipine (IV) | 66 |
| Nifedipine (oral) | 70 |
| Nimodipine (oral) | 4 |
| Nitroglycerin | |
| Nitroglycerin (IV) | 37 |
| Nitroglycerin (oral) | 95 |
| Prazosin (oral) | 20 |
| Propranolol (oral) | 64 |
| Spironolactone (oral) | 97 |
| Valsartan (oral) | 18 |
| Verapamil (oral) | 54 |

Table S3. Baseline characteristics of metoprolol, amlodipine and carvedilol administered following severe inpatient hypertension developing

|  | Treated with oral metoprolol only N=998 | Treated with oral amlodipine only N=659 | Treated with oral carvedilol only N=473 |
| --- | --- | --- | --- |
| *Dose* | *25 [25, 50]* | *5 [5, 10]* | *12.5 [6.25, 25]* |
| **Demographics** | | | |
| Age, years | 75.5 (13.7) | 71.9 (15.6) | 71.7 (14.3) |
| Male | 441 (44.2) | 295 (44.8) | 227 (48.0) |
| Black | 158 (15.8) | 184 (27.9) | 120 (25.4) |
| Hispanic or Latino | 66 (6.6) | 76 (11.5) | 72 (15.2) |
| Admitted to surgical ward | 145 (14.5) | 80 (12.1) | 52 (11.0) |
| CVD admissions* | 494 (49.5) | 176 (26.7) | 300 (63.4) |
| **Comorbidities** | | | |
| Congestive Heart Failure | 381 (38.2) | 151 (22.9) | 291 (22.9) |
| Cardiac Arrhythmia | 551 (55.2) | 234 (35.5) | 245 (35.4) |
| Valvular Disease | 271 (27.2) | 106 (16.1) | 166 (15.9) |
| Pulmonary Circulation Disorder | 153 (15.3) | 75 (11.4) | 85 (11.3) |
| Peripheral Vascular Disease | 325 (32.6) | 151 (22.9) | 210 (22.9) |
| Hypertension | 865 (86.7) | 548 (83.2) | 445 (83.1) |
| Paralysis | 60 (6.0) | 30 (4.6) | 26 (4.6) |
| Other Neurological Disorder | 315 (31.6) | 198 (30.0) | 151 (29.9) |
| Chronic Pulmonary Disorders | 390 (39.1) | 240 (36.4) | 211 (36.5) |
| Diabetes | 445 (44.6) | 272 (41.3) | 304 (41.2) |
| Hypothyroidism | 230 (23.0) | 149 (22.6) | 102 (22.7) |
| Renal Failure | 357 (35.8) | 220 (33.4) | 278 (33.4) |
| Liver Disease | 118 (11.8) | 115 (17.5) | 75 (17.6) |
| Peptic Ulcer Disease (no bleeding) | 62 (6.2) | 47 (7.1) | 32 (7.2) |
| AIDS/HIV | 6 (0.6) | 18 (2.7) | 4 (2.7) |
| Malignancy | 207 (20.7) | 147 (22.3) | 86 (22.4) |
| Rheumatoid Arthritis/Collagen Disorders | 79 (7.9) | 46 (7.0) | 49 (7.0) |
| Coagulopathy | 140 (14.0) | 89 (13.5) | 73 (13.6) |
| Obesity | 272 (27.2) | 138 (20.9) | 151 (20.9) |
| Weight Loss | 197 (19.7) | 135 (20.5) | 97 (20.5) |
| Fluid and Electrolyte Disorders | 580 (58.1) | 351 (53.3) | 325 (53.1) |
| Blood Loss Anemia | 81 (8.1) | 55 (8.3) | 56 (8.2) |
| Iron Deficiency Anemia | 193 (19.3) | 148 (22.5) | 145 (22.4) |
| Alcohol Use Disorder | 106 (10.6) | 116 (17.6) | 57 (17.6) |
| Drug Abuse | 112 (11.2) | 114 (17.3) | 73 (17.4) |
| Psychosis | 50 (5.0) | 54 (8.2) | 24 (8.2) |
| Depression | 350 (35.1) | 230 (34.9) | 189 (34.8) |
| *Elixhauser Score* | 7.0 [4.0, 10.0] | 6.0 [3.0, 9.0] | 8.0 [6.0, 11.0] |

Table S3 (Continued)

|  | | | Treated with oral metoprolol only N=998 | | | Treated with oral amlodipine only N=659 | Treated with oral carvedilol only N=473 | | |
| --- | --- | --- | --- | --- | --- | --- | --- | --- | --- |
| **Admission Characteristics, Median [IQR]** | | | | | | | | | |
| MAP | | | 102.0 [89.0, 113.0] | | | 105.0 [93.0, 115.0] | 100.7 [87.3, 111.7] | | |
| SBP (mmHg) | | | 148.0 [130.0, 166.0] | | | 152.0 [136.0, 170.0] | 148.0 [130.0, 165.0] | | |
| DBP (mmHg) | | | 78.0 [66.0, 90.0] | | | 79.0 [69.0, 90.0] | 75.0 [64.0, 87.5] | | |
| Heart Rate (bpm) | | | 84.0 [70.0, 99.0] | | | 83.0 [70.0, 96.0] | 79.0 [69.5, 91.0] | | |
| BMI (kg/m^2^) | | | 27.5 [23.2, 32.3] | | | 26.5 [22.4, 31.5] | 28.2 [24.5, 33.7] | | |
| **BP at time of incident severe inpatient hypertension** | | | | | | | | | |
| MAP | | | 121.7 [114.7, 127.3] | | | 121.3 [115.5, 127.7] | 119.7 [114.0, 126.33] | | |
| SBP | | | 185.0 [181.0, 189.0] | | | 185.0 [182.0, 190.0] | 185.0 [182.0, 189.0] | | |
| DBP | | | 88.0 [79.0, 101.0] | | | 88.0 [80.0, 98.0] | 85.0 [78.0, 97.0] | | |
| **Coefficient of variation (CV) of BP and MAP before onset of severe inpatient hypertension (SD/mean)** | | | | | | | | | |
| MAP CV | | | 0.10 [0.07, 0.13] | | | 0.09 [0.07, 0.13] | 0.11 [0.08, 0.14] | | |
| SBP CV | | | 0.11 [0.08, 0.14] | | | 0.10 [0.07, 0.14] | 0.11 [0.08, 0.15] | | |
| DBP CV | | | 0.13 [0.09, 0.17] | | | 0.11 [0.08, 0.15] | 0.13 [0.09, 0.17] | | |
| **Admission Laboratory Values Median [IQR]** | | | | | | | | | |
| Serum Sodium (meq/L) | | | 139.0 [136.0, 141.0] | | | 139.0 [136.0, 142.0] | 138.0 [135.0, 141.0] | | |
| Serum Potassium (meq/L) | | | 4.2 [3.9, 4.6] | | | 4.2 [3.80, 4.60] | 4.3 [3.9, 4.9] | | |
| Serum Chloride (meq/L) | | | 102.0 [98.0, 105.0] | | | 101.0 [98.0, 105.0] | 101.0 [97.0, 104.0] | | |
| Serum Bicarbonate (meq/L) | | | 25.0 [22.0, 27.0] | | | 24.0 [21.1, 27.0] | 24.0 [21.0, 27.0] | | |
| BUN (mg/dL) | | | 22.0 [15.0, 34.0] | | | 23.0 [15.0, 37.0] | 29.0 [18.0, 46.0] | | |
| Serum Creatinine (mg/dL) | | | 1.1 [0.8, 1.6] | | | 1.0 [0.8, 1.7] | 1.5 [1.0, 2.8] | | |
| eGFR (ml/min/1.73m2 ) | | | 57.9 [37.4, 83.4] | | | 62.5 [34.8, 89.9] | 41.7 [19.8, 66.9] | | |
| White Blood Cell Count (x1000/uL) | | | 9.2 [6.8, 12.3] | | | 8.9 [6.5, 12.2] | 9.0 [6.8, 11.5] | | |
| Platelet Count (x1000/uL) | | | 227.0 [177.0, 289.0] | | | 230.0 [181.0, 297.3] | 211.5 [163.0, 272.0] | | |
| Hemoglobin, g/dl | | | 11.9 [10.5, 13.5] | | | 11.9 [10.4, 13.5] | 11.2 [9.8, 12.6] | | |
| Hematocrit, % | | | 36.9 [32.7, 41.1] | | | 36.7 [32.3, 41.2] | 34.7 [30.8, 38.7] | | |
| **Received any of the following medications 0-6 hours before time of incident severe inpatient hypertension** | | | | | | | | |  |
| Steroids | 36 (3.6) | | | | 20 (3.0) | | | 22 (4.7) |  |
| NSAID | 16 (1.6) | | | | 21 (3.2) | | | 3 (0.6) |  |
| Crystalloid | 140 (14.0) | | | | 101 (15.3) | | | 65 (13.7) |  |
| Narcotic | 146 (14.6) | | | | 105 (15.9) | | | 71 (15.0) |  |
| Sedatives | 52 (5.2) | | | | 59 (9.0) | | | 23 (4.9) |  |
| **Number of BP measurements available following treatment with each of the relevant medications** | | | | | | | | |  |
| Number of measurements | | 1 [1, 2] | | 1 [1, 2] | | | | 1 [1, 2] |  |
| **Lowest BP measured after 6 hours from severe inpatient HTN development** | | | | | | | | |  |
| MAP | | 102.0 [92.0, 111.7] | | 105.3 [96.8, 114.3] | | | | 102.3 [91.3, 110.0] |  |
| SBP | | 152.0 [136.2, 168.0] | | 159.0 [143.0, 174.0] | | | | 153.0 [136.0, 169.0] |  |
| DBP | | 74.0 [66.0, 83.0] | | 76.0 [68.0, 85.5] | | | | 73.0 [66.0, 81.0] |  |

Values are presented as count (percent) or median (IQR). BMI: body mass index, BP: blood pressure, MAP: mean arterial pressure; SBP: systolic BP; DBP: diastolic BP; bpm: beats per minute, BUN: Blood Urea Nitrogen. eGFR: Estimated glomerular filtration rate
* CVD (cardiovascular) admissions: cerebrovascular accident, myocardial infarction, heart failure or abdominal aortic aneurysm (based on the following ICD-10 codes: I60, I61, I63, I64, G45, I62, I66, I67, I68, G46 , I-21, I22, I25.2, I71)
Table S4. Baseline characteristics of oral and IV hydralazine administered following severe inpatient hypertension developing

|  | Treated with IV hydralazine only  N=632 | Treated with oral hydralazine only N=390 |
| --- | --- | --- |
| *Dose* | *10 [5, 10]* | *25 [25, 50]* |
| **Demographics** | | |
| Age, years | 70.9 (16.7) | 72.1 (15.4) |
| Male | 271 (42.3) | 170 (43.6) |
| Black | 145 (22.9) | 118 (30.3) |
| Hispanic or Latino | 66 (10.4) | 50 (12.8) |
| Admitted to surgical ward | 180 (28.5) | 40 (10.3) |
| CVD admissions* | 223 (35.3) | 182 (46.7) |
| **Comorbidities** | | |
| Congestive Heart Failure | 164 (25.9) | 172 (441) |
| Cardiac Arrhythmia | 232 (36.7) | 180 (46.2) |
| Valvular Disease | 119 (18.8) | 106 (27.2) |
| Pulmonary Circulation Disorder | 68 (10.8) | 71 (18.2) |
| Peripheral Vascular Disease | 140 (22.2) | 136 (34.9) |
| Hypertension | 503 (79.6) | 356 (91.3) |
| Paralysis | 30 (4.7) | 15 (3.8) |
| Other Neurological Disorder | 165 (26.1) | 122 (31.3) |
| Chronic Pulmonary Disorders | 189 (29.9) | 164 (42.1) |
| Diabetes | 264 (41.8) | 222 (56.9) |
| Hypothyroidism | 134 (21.2) | 102 (26.2) |
| Renal Failure | 207 (32.8) | 227 (58.2) |
| Liver Disease | 93 (14.7) | 63 (16.2) |
| Peptic Ulcer Disease (no bleeding) | 33 (5.2) | 38 (9.7) |
| AIDS/HIV | 8 (1.3) | 2 (0.5) |
| Malignancy | 139 (22.0) | 73 (18.7) |
| Rheumatoid Arthritis/Collagen Disorders | 52 (8.2) | 35 (9.0) |
| Coagulopathy | 83 (13.1) | 73 (18.7) |
| Obesity | 138 (21.8) | 134 (34.4) |
| Weight Loss | 122 (19.3) | 82 (21.0) |
| Fluid and Electrolyte Disorders | 330 (52.2) | 269 (69.0) |
| Blood Loss Anemia | 40 (6.3) | 50 (12.8) |
| Iron Deficiency Anemia | 128 (20.3) | 112 (28.7) |
| Alcohol Use Disorder | 102 (16.1) | 50 (12.8) |
| Drug Abuse | 98 (15.5) | 70 (17.9) |
| Psychosis | 36 (5.7) | 12 (5.9) |
| Depression | 213 (33.7) | 155 (39.7) |
| *Elixhauser Score* | 6.0 [3.0, 9.0] | 8.0 [5.0, 11.0] |

Table S4 (Continued)

|  | Treated with IV hydralazine only  N=632 | | | Treated with oral hydralazine only N=390 |
| --- | --- | --- | --- | --- |
| **Admission Characteristics, Median [IQR]** | | | | |
| MAP | 109.5 [98.3, 120.0] | | | 103.0 [92.7, 113.3] |
| SBP (mmHg) | 160.0 [141.0, 180.0] | | | 156.0 [138.0, 170.0] |
| DBP (mmHg) | 82.5 [73.0, 93.3] | | | 78.0 [66.0, 88.0] |
| Heart Rate (bpm) | 78.0 [68.0, 93.0] | | | 78.0 [68.0, 88.0] |
| BMI (kg/m^2^) | 26.7 [22.4, 31.2] | | | 27.6 [23.3, 32.4] |
| **BP at time of incident severe inpatient hypertension** | | | | |
| MAP | 123.7 [117.3, 131.3] | | | 119.7 [113.1, 127.7] |
| SBP | 188.0 [183.0, 196.0] | | | 186.0 [183.0, 190.8] |
| DBP | 90.0 [81.0, 101.0] | | | 86.0 [79.0, 97.0] |
| **Coefficient of variation (CV) of BP and MAP before onset of severe inpatient hypertension (SD/mean)** | | | | |
| MAP CV | 0.10 [0.67, 0.13] | | | 0.09 [0.06, 0.12] |
| SBP CV | 0.10 [0.07, 0.14] | | | 0.10 [0.07, 0.14] |
| DBP CV | 0.13 [0.11, 0.15] | | | 0.12 [0.08, 0.15] |
| **Admission Laboratory Values Median [IQR]** | | | | |
| Serum Sodium (meq/L) | 139.0 [136.0, 142.0] | | | 139.0 [135.0, 141.0] |
| Serum Potassium (meq/L) | 4.1 [3.8, 4.5] | | | 4.3 [3.9, 4.8] |
| Serum Chloride (meq/L) | 102.0 [98.0, 105.0] | | | 101.0 [97.0, 105.0] |
| Serum Bicarbonate (meq/L) | 25.0 [22.0, 27.0] | | | 24.0 [21.0, 26.0] |
| BUN (mg/dL) | 20.0 [14.0, 30.0] | | | 28.5 [18.0, 46.0] |
| Serum Creatinine (mg/dL) | 1.09 [0.80, 1.55] | | | 1.50 [1.05, 2.82] |
| eGFR (ml/min/1.73m2 ) | 62.8 [38.4, 89.9] | | | 39.2 [21.4, 64.5] |
| White Blood Cell Count (x1000/uL) | 8.8 [6.5, 11.5] | | | 7.8 [5.9, 10.7] |
| Platelet Count (x1000/uL) | 219.0 [172.0, 279.0] | | | 225.0 [169.3, 274.3] |
| Hemoglobin, g/dl | 12.0 [10.4, 13.4] | | | 11.3 [9.5, 12.5] |
| Hematocrit, % | 36.8 [32.3, 40.9] | | | 34.9 [29.9, 38.6] |
| **Received any of the following medications 0-6 hours before time of incident severe inpatient hypertension** | | | | |
| Steroids | | 28 (4.4) | 21 (5.4) | |
| NSAID | | 16 (2.5) | 6 (1.5) | |
| Crystalloid | | 116 (18.4) | 51 (13.1) | |
| Narcotic | | 117 (18.5) | 70 (17.9) | |
| Sedatives | | 35 (5.5) | 17 (4.4) | |
| **Number of BP measurements available following treatment with each of the relevant medications** | | | | |
| Number of measurements | | 1 [1, 1] | 1 [1, 1] | |
| **Lowest BP measured after 6 hours from severe inpatient HTN development** | | | | |
| MAP | | 102.3 [92.0, 111.0] | 106.0 [96.0, 113.7] | |
| SBP | | 156.0 [141.0, 169.0] | 163.0 [147.2, 176.0] | |
| DBP | | 72.0 [64.0, 81.0] | 74.0 [66.0, 83.0] | |

Values are presented as count (percent) or median (IQR). BMI: body mass index, BP: blood pressure, MAP: mean arterial pressure; SBP: systolic BP; DBP: diastolic BP; bpm: beats per minute, BUN: Blood Urea Nitrogen. eGFR: Estimated glomerular filtration rate
* CVD (cardiovascular) admissions: cerebrovascular accident, myocardial infarction, heart failure or abdominal aortic aneurysm (based on the following ICD-10 codes: I60, I61, I63, I64, G45, I62, I66, I67, I68, G46 , I-21, I22, I25.2, I71)
**Note that all covariates are different significantly different across the five common antihypertensives except for sex, steroids, narcotics, paralysis, other neurological disorders, hypothyroidism, malignancy, rheumatoid arthritis, coagulopathy, weight loss, psychosis.

Table S5. Association of the most common antihypertensives given for treatment of severe inpatient hypertension with blood pressure among patients admitted to the medical ward only

| β (95%CI) | Model 1 | Model 2 | Model 3 |
| --- | --- | --- | --- |
| **Treated with oral metoprolol vs. untreated** | | | |
| Slope of MAP | 3.9 [1.31, 6.54] | 5.2 [2.59, 7.89] | 5.8 [3.12, 8.48] |
| Slope of SBP | 5.0 [1.08, 8.96] | 5.5 [1.54, 9.53] | 6.5 [2.45, 10.64] |
| Slope of DBP | 3.1 [0.50, 5.67] | 4.9 [2.40, 7.54] | 5.0 [2.45, 7.61] |
| **Treated with oral amlodipine vs. untreated** | | | |
| Slope of MAP | 0.3 [-3.21, 3.90] | 0.9 [-2.75, 4.49] | 0.8 [-2.85, 4.463.] |
| Slope of SBP | -13.6 [-18.50, -8.70] | -13.8 [-18.85, -8.77] | -14.6 [-19.67, -9.46] |
| Slope of DBP | 7.7 [3.91, 11.42] | 8.1 [4.37,11.86] | 8.2 [4.35, 12.07] |
| **Treated with oral carvedilol vs. untreated** | | | |
| Slope of MAP | -10.6 [-13.41, -7.72] | -9.8 [-12.67, -6.90] | -9.4 [-12.29, -6.58] |
| Slope of SBP | -5.5 [-9.64, -1.38] | -6.9 [-11.17, -2.75] | -5.4 [-9.08, -1.71] |
| Slope of DBP | -12.9 [-15.77. -10.01] | -10.9 [-13.80, -8.14] | -11.1 [-13.95, -8.31] |
| **Treated with IV hydralazine vs. untreated** | | | |
| Slope of MAP | -9.9 [-13.05, -6.64] | -9.0 [-12.36, -5.69] | -8.5 [-12.63, -4.28] |
| Slope of SBP | -12.5 [-17.07, -7.84] | -12.9 [-17.68, -8.04] | -15.4 [-21.29, -9.47] |
| Slope of DBP | -9.1 [-12.32, -5.97] | -8.3 [-11.58, -4.98] | -6.4 [-10.59, -2.16] |
| **Treated with oral hydralazine vs. untreated** | | | |
| Slope of MAP | -9.9 [-15.33, -4.45] | -9.3 [14.63, -3.86] | -9.4 [-14.87, -3.99] |
| Slope of SBP | -14.2 [-21.97, -6.35] | -14.4 [-22.31, -6.51] | -13.4 [-21.45, -5.49] |
| Slope of DBP | -8.1 [-13.73, -2.49] | -7.2 [-12.67, -1.79] | -8.1 [-13.70, -2.46] |

BP: blood pressure; IV: intravenous; MAP: mean arterial pressure; SBP: systolic blood pressure; DBP: diastolic blood pressure
*Within 6 hours of developing severe inpatient HTN, number of patients that were treated with only: oral metoprolol: 623; oral amlodipine: 442; oral carvedilol:296; IV hydralazine: 354; oral hydralazine: 354 vs. untreated (7,955)*

**Model 1**: unadjusted
**Model 2**: age, sex, race, ethnicity, ward, comorbidities (congestive heart failure, cardiac arrythmia, peripheral vascular disease, hypertension, diabetes, hypothyroidism, renal failure, AIDS/HIV, cancer, alcohol abuse, drug abuse, psychosis, depression), baseline laboratory values (sodium, potassium, chloride, bicarbonate, BUN, eGFR, WBCC, platelet count, hemoglobin, hematocrit), NSAID use 0-6 hours before time of severe inpatient HTN, crystalloid use 0-6 hours before time of severe inpatient HTN, steroid use 0-6 hours before time of severe inpatient HTN, narcotic use 0-6 hours before time of severe inpatient HTN, sedative use 0-6 hours before time of severe inpatient HTN, hospital
**Model 3**: age, sex, race, ethnicity, comorbidities (congestive heart failure, cardiac arrythmia, valvular disease, pulmonary circulation disorder, peripheral vascular disease, hypertension, paralysis, other neurological disorders, chronic pulmonary disease, diabetes, hypothyroidism, renal failure, liver disease, peptic ulcer disease excluding bleeding, AIDS/HIV, lymphoma, cancer, rheumatoid arthritis/collagen disorder, coagulopathy, obesity, weight loss, fluid and electrolyte disorders, blood loss anemia, deficiency anemia, alcohol abuse, drug abuse, psychosis, depression), baseline laboratory values (sodium, potassium, chloride, bicarbonate, BUN, eGFR, WBCC, platelet count, hemoglobin, hematocrit), NSAID use 0-6 hours before time of severe inpatient HTN, crystalloid use 0-6 hours before time of severe inpatient HTN, steroid use 0-6 hours before time of severe inpatient HTN, narcotic use 0-6 hours before time of severe inpatient HTN, sedative use 0-6 hours before time of severe inpatient HTN, maximum MAP before time of severe inpatient HTN development, minimum MAP before time of severe inpatient HTN development, coefficient of variation of MAP before time of severe inpatient HTN development.
hospital

Table S6. Association of the most common antihypertensives given for treatment* of severe inpatient hypertension with blood pressure

| β (95%CI) | Model 1 | Model 2 | Model 3 |
| --- | --- | --- | --- |
| **Treated with oral metoprolol vs. untreated** | | | |
| Slope of MAP | 7.0 [3.69, 10.45] | 8.1 [4.64, 11.60] | 9.6 [5.97, 13.24] |
| Slope of SBP | 4.59 [-0.43, 9.62] | 5.2 [-0.03, 10.36] | 7.3 [2.03, 12.70] |
| Slope of DBP | 7.9 [4.67, 11.27] | 9.4 [6.04, 12.76] | 10.1 [6.73, 13.84] |
| **Treated with oral amlodipine vs. untreated** | | | |
| Slope of MAP | 2.1 [-1.86, 6.01] | 2.5 [-1.53, 6.51] | 2.9 [-118, 6.99] |
| Slope of SBP | -14.1 [-19.47, -8.63] | -14.6 [-20.19, -8.95] | -13.4 [-19.47, -8.04] |
| Slope of DBP | 11.1 [6.93, 15.30] | 11.6 [7.44, 15.76] | 11.1 [6.73, 15.34] |
| **Treated with oral carvedilol vs. untreated** | | | |
| Slope of MAP | 9.6 [2.78, 16.36] | 11.8 [4.63, 19.06] | 11.1 [4.09, 18.54] |
| Slope of SBP | 29.0 [18.02, 39.68] | 30.4 [18.80, 41.94] | 31.1 [19.36, 42.84] |
| Slope of DBP | 0.7 [-4.03, 5.43] | 2.9 [-2.20, 8.11] | 2.1 [-2.72, 7.03] |
| **Treated with IV hydralazine vs. untreated** | | | |
| Slope of MAP | -12.9 [-15.43, -10.49] | -11.2 [-13.66, -8.74] | -11.5 [-14.51, -8.43] |
| Slope of SBP | -15.1 [-18.72, -11.55] | -15.5 [-19.13, -11.80] | -18.9 [-23.20, -14.66] |
| Slope of DBP | -11.8 [-14.26, -9.39] | -12.2 [-14.73, -9.72] | -13.6 [-16.56, -10.58] |
| **Treated with oral hydralazine vs. untreated** | | | |
| Slope of MAP | -11.0 [-16.74, -5.27] | -10.4 [-16.18, -4.66] | -9.9 [-15.97, -3.91] |
| Slope of SBP | -13.2 [-21.64, -4.70] | -13.7 [-22.23, -5.11] | -12.6 [-21.31, -3.93] |
| Slope of DBP | -9.5 [-15.36, -3.64] | -8.5 [14.31, -2.69] | -9.0 [-15.31, -2.72] |

BP: blood pressure; IV: intravenous; MAP: mean arterial pressure; SBP: systolic blood pressure; DBP: diastolic blood pressure
*** treatment: receiving a new antihypertensive medication (medication not prescribed/day ≥90% of their hospital stay)**
*Within 6 hours of developing severe inpatient HTN, number of patients that were treated with only: oral metoprolol: 283; oral amlodipine: 293; oral carvedilol:102; IV hydralazine: 553; oral hydralazine: 198 vs. untreated (13,753)*

**Model 1**: unadjusted
**Model 2**: age, sex, race, ethnicity, ward, comorbidities (congestive heart failure, cardiac arrythmia, peripheral vascular disease, hypertension, diabetes, hypothyroidism, renal failure, AIDS/HIV, cancer, alcohol abuse, drug abuse, psychosis, depression), baseline laboratory values (sodium, potassium, chloride, bicarbonate, BUN, eGFR, WBCC, platelet count, hemoglobin, hematocrit), NSAID use 0-6 hours before time of severe inpatient HTN, crystalloid use 0-6 hours before time of severe inpatient HTN, steroid use 0-6 hours before time of severe inpatient HTN, narcotic use 0-6 hours before time of severe inpatient HTN, sedative use 0-6 hours before time of severe inpatient HTN, hospital
**Model 3**: age, sex, race, ethnicity, ward, comorbidities (congestive heart failure, cardiac arrythmia, valvular disease, pulmonary circulation disorder, peripheral vascular disease, hypertension, paralysis, other neurological disorders, chronic pulmonary disease, diabetes, hypothyroidism, renal failure, liver disease, peptic ulcer disease excluding bleeding, AIDS/HIV, lymphoma, cancer, rheumatoid arthritis/collagen disorder, coagulopathy, obesity, weight loss, fluid and electrolyte disorders, blood loss anemia, deficiency anemia, alcohol abuse, drug abuse, psychosis, depression), baseline laboratory values (sodium, potassium, chloride, bicarbonate, BUN, eGFR, WBCC, platelet count, hemoglobin, hematocrit), NSAID use 0-6 hours before time of severe inpatient HTN, crystalloid use 0-6 hours before time of severe inpatient HTN, steroid use 0-6 hours before time of severe inpatient HTN, narcotic use 0-6 hours before time of severe inpatient HTN, sedative use 0-6 hours before time of severe inpatient HTN, maximum MAP before time of severe inpatient HTN development, minimum MAP before time of severe inpatient HTN development, coefficient of variation of MAP before time of severe inpatient HTN development.
hospital

Table S7. Association of the most common antihypertensives given for treatment of severe inpatient hypertension with blood pressure among non-cardiovascular disease admissions

| β (95%CI) | Model 1 | Model 2 | Model 3 |
| --- | --- | --- | --- |
| **Treated with oral metoprolol vs. untreated** | | | |
| Slope of MAP | 5.6 [2.85, 8.30] | 5.8 [3.08, 8.57] | 6.8 [3.89, 9.72] |
| Slope of SBP | 11.6 [7.47, 15.72] | 10.8 [6.63, 14.94] | 13.3 [8.84, 17.77] |
| Slope of DBP | 2.2 [-0.45, 4.76] | 2.1 [0.52, 5.75] | 3.1 [0.38, 5.89] |
| **Treated with oral amlodipine vs. untreated** | | | |
| Slope of MAP | 1.7 [-1.62, 5.02] | 1.7 [-1.74, 5.12] | 1.3 [-2.28, 4.86] |
| Slope of SBP | -11.7 [-16.34, -6.96] | -13.1 [-18.03, -8.19] | -14.6 [-19.63, -9.48] |
| Slope of DBP | 8.6 [5.21, 11.99] | 9.2 [5.61, 12.69] | 9.1 [5.43, 12.80] |
| **Treated with oral carvedilol vs. untreated** | | | |
| Slope of MAP | -4.4 [-12.14, 3.29] | -3.8 [-12.45, 4.77] | -1.8 [-11.12, 7.60] |
| Slope of SBP | -2.15 [-13.47, 9.17] | -4.2 [-16.94, 8.63] | -2.4 [-16.29, 1.15] |
| Slope of DBP | -5.0 [-12.49, 2.46] | -3.0 [-11.23, 5.17] | -1.3[-10.26, 7.69] |
| **Treated with IV hydralazine vs. untreated** | | | |
| Slope of MAP | -11.8 [-14.62, -8.89] | -10.9 [-13.83, -8.03] | -10.4 [-13.81, -7.01] |
| Slope of SBP | -14.7 [-18.92, -10.53] | -14.6 [-18.92, -10.36] | -17.0 [-22.04, -12.02] |
| Slope of DBP | -10.5 [-13.29, -7.75] | -9.9 [-12.68, -7.09] | -8.5 [-11.83, -5.15] |
| **Treated with oral hydralazine vs. untreated** | | | |
| Slope of MAP | -7.6 [-13.14, -1.99] | -6.7 [12.31, -1.09] | -6.8 [-12.58, -1.03] |
| Slope of SBP | -6.3 [-17.29, -1.08] | -9.7 [-18.12, -1.28] | -6.9 [-15.69, 1.79] |
| Slope of DBP | -3.1 [-12.38, -0.90] | -5.6 [-11.31, 0.13] | -7.0 [-12.94, -1.11] |

BP: blood pressure; IV: intravenous; MAP: mean arterial pressure; SBP: systolic blood pressure; DBP: diastolic blood pressure
*Within 6 hours of developing severe inpatient HTN, number of patients that were treated with only: oral metoprolol: 504; oral amlodipine: 483; oral carvedilol:173; IV hydralazine: 409; oral hydralazine: 208 vs. untreated (9,258)*

**Model 1**: unadjusted
**Model 2**: age, sex, race, ethnicity, ward, comorbidities (congestive heart failure, cardiac arrythmia, peripheral vascular disease, hypertension, diabetes, hypothyroidism, renal failure, AIDS/HIV, cancer, alcohol abuse, drug abuse, psychosis, depression), baseline laboratory values (sodium, potassium, chloride, bicarbonate, BUN, eGFR, WBCC, platelet count, hemoglobin, hematocrit), NSAID use 0-6 hours before time of severe inpatient HTN, crystalloid use 0-6 hours before time of severe inpatient HTN, steroid use 0-6 hours before time of severe inpatient HTN, narcotic use 0-6 hours before time of severe inpatient HTN, sedative use 0-6 hours before time of severe inpatient HTN, hospital
**Model 3**: age, sex, race, ethnicity, ward, comorbidities (congestive heart failure, cardiac arrythmia, valvular disease, pulmonary circulation disorder, peripheral vascular disease, hypertension, paralysis, other neurological disorders, chronic pulmonary disease, diabetes, hypothyroidism, renal failure, liver disease, peptic ulcer disease excluding bleeding, AIDS/HIV, lymphoma, cancer, rheumatoid arthritis/collagen disorder, coagulopathy, obesity, weight loss, fluid and electrolyte disorders, blood loss anemia, deficiency anemia, alcohol abuse, drug abuse, psychosis, depression), baseline laboratory values (sodium, potassium, chloride, bicarbonate, BUN, eGFR, WBCC, platelet count, hemoglobin, hematocrit), NSAID use 0-6 hours before time of severe inpatient HTN, crystalloid use 0-6 hours before time of severe inpatient HTN, steroid use 0-6 hours before time of severe inpatient HTN, narcotic use 0-6 hours before time of severe inpatient HTN, sedative use 0-6 hours before time of severe inpatient HTN, maximum MAP before time of severe inpatient HTN development, minimum MAP before time of severe inpatient HTN development, coefficient of variation of MAP before time of severe inpatient HTN development.
hospital

Table S8. Association of intravenous and oral labetalol following severe inpatient hypertension development with blood pressure

| β (95%CI) | Model 1 | Model 2 | Model 3 |
| --- | --- | --- | --- |
| **Treated with IV labetalol vs. untreated** | | | |
| Slope of MAP | -9.4 [-12.08, -6.77] | -9.42 [-12.12, -6.71] | -7.59 [-11.78, -3.26] |
| Slope of SBP | -16.2 [-20.05, -12.36] | -17.1 [-20.98, -13.13] | -16.2 [-22.28, -10.04] |
| Slope of DBP | -6.7 [-9.33, -4.06] | -6.29 [-8.97, -3.62] | -5.6 [-9.95, -1.26] |
| **Treated with oral labetalol vs. untreated** | | | |
| Slope of MAP | -4.5 [-10.52, 1.58] | -4.2 [-10.16, 1.75] | -4.5 [-10.19, 1.96] |
| Slope of SBP | 2.3 [-6.40, 10.95] | 2.3 [-6.43, 11.08] | 2.1 [-7.32, 11.56] |
| Slope of DBP | -3.2 [-13.2, -0.88] | -6.8 [-12.75, -0.86] | -7.2 [-13.86, -0.59] |

BP: blood pressure; IV: intravenous; MAP: mean arterial pressure; SBP: systolic blood pressure; DBP: diastolic blood pressure
*Within 6 hours of developing severe inpatient HTN, number of patients that were treated with only: IV Labetalol: 337 and oral Labetalol: 129 and untreated: 13,753
IV labetalol dose: 10 [10, 20] and Oral labetalol dose: 100 [100, 200]*

**Model 1**: unadjusted
**Model 2**: age, sex, race, ethnicity, ward, comorbidities (congestive heart failure, cardiac arrythmia, peripheral vascular disease, hypertension, diabetes, hypothyroidism, renal failure, AIDS/HIV, cancer, alcohol abuse, drug abuse, psychosis, depression), baseline laboratory values (sodium, potassium, chloride, bicarbonate, BUN, eGFR, WBCC, platelet count, hemoglobin, hematocrit), NSAID use 0-6 hours before time of severe inpatient HTN, crystalloid use 0-6 hours before time of severe inpatient HTN, steroid use 0-6 hours before time of severe inpatient HTN, narcotic use 0-6 hours before time of severe inpatient HTN, sedative use 0-6 hours before time of severe inpatient HTN, hospital
**Model 3**: age, sex, race, ethnicity, ward, comorbidities (congestive heart failure, cardiac arrythmia, valvular disease, pulmonary circulation disorder, peripheral vascular disease, hypertension, paralysis, other neurological disorders, chronic pulmonary disease, diabetes, hypothyroidism, renal failure, liver disease, peptic ulcer disease excluding bleeding, AIDS/HIV, lymphoma, cancer, rheumatoid arthritis/collagen disorder, coagulopathy, obesity, weight loss, fluid and electrolyte disorders, blood loss anemia, deficiency anemia, alcohol abuse, drug abuse, psychosis, depression), baseline laboratory values (sodium, potassium, chloride, bicarbonate, BUN, eGFR, WBCC, platelet count, hemoglobin, hematocrit), NSAID use 0-6 hours before time of severe inpatient HTN, crystalloid use 0-6 hours before time of severe inpatient HTN, steroid use 0-6 hours before time of severe inpatient HTN, narcotic use 0-6 hours before time of severe inpatient HTN, sedative use 0-6 hours before time of severe inpatient HTN, maximum MAP before time of severe inpatient HTN development, minimum MAP before time of severe inpatient HTN development, coefficient of variation of MAP before time of severe inpatient HTN development.
hospital
