# Supplementary figures and images for: Blood pressure response to commonly administered antihypertensives for severe inpatient hypertension
Source: PLoS One. 2022 Apr 6;17(4):e0265497. doi: 10.1371/journal.pone.0265497 (PMC8985959; doi:10.1371/journal.pone.0265497)

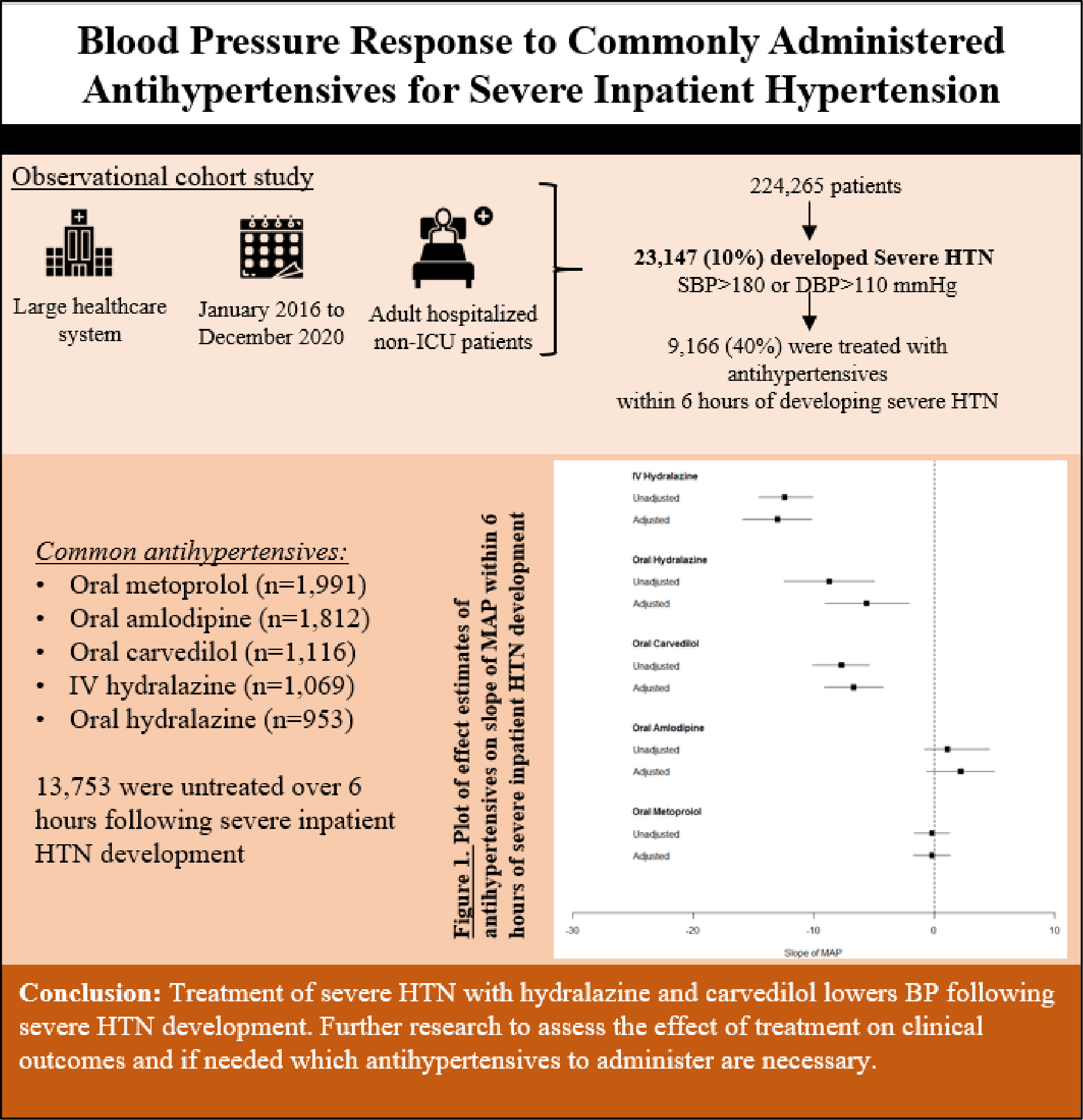

Supplement: S1 Graphical abstract — (TIF) [file pone.0265497.s002.tif]
